# Transcriptomic Immune Response of *Tenebrio molitor* Pupae to Parasitization by *Scleroderma guani*

**DOI:** 10.1371/journal.pone.0054411

**Published:** 2013-01-14

**Authors:** Jia-Ying Zhu, Pu Yang, Zhong Zhang, Guo-Xing Wu, Bin Yang

**Affiliations:** 1 Key Laboratory of Forest Disaster Warning and Control of Yunnan Province, Southwest Forestry University, Kunming, China; 2 Research Institute of Resource Insects, Chinese Academy of Forestry, Kunming, China; 3 Department of Pathogen Biology, Taishan Medical University, Tai'an, China; 4 College of Plant Protection, Yunnan Agricultural University, Kunming, China; Volcani Center, Israel

## Abstract

**Background:**

Host and parasitoid interaction is one of the most fascinating relationships of insects, which is currently receiving an increasing interest. Understanding the mechanisms evolved by the parasitoids to evade or suppress the host immune system is important for dissecting this interaction, while it was still poorly known. In order to gain insight into the immune response of *Tenebrio molitor* to parasitization by *Scleroderma guani*, the transcriptome of *T. molitor* pupae was sequenced with focus on immune-related gene, and the non-parasitized and parasitized *T. molitor* pupae were analyzed by digital gene expression (DGE) analysis with special emphasis on parasitoid-induced immune-related genes using Illumina sequencing.

**Methodology/Principal Findings:**

In a single run, 264,698 raw reads were obtained. *De novo* assembly generated 71,514 unigenes with mean length of 424 bp. Of those unigenes, 37,373 (52.26%) showed similarity to the known proteins in the NCBI nr database. Via analysis of the transcriptome data in depth, 430 unigenes related to immunity were identified. DGE analysis revealed that parasitization by *S. guani* had considerable impacts on the transcriptome profile of *T. molitor* pupae, as indicated by the significant up- or down-regulation of 3,431 parasitism-responsive transcripts. The expression of a total of 74 unigenes involved in immune response of *T. molitor* was significantly altered after parasitization.

**Conclusions/Significance:**

obtained *T. molitor* transcriptome, in addition to establishing a fundamental resource for further research on functional genomics, has allowed the discovery of a large group of immune genes that might provide a meaningful framework to better understand the immune response in this species and other beetles. The DGE profiling data provides comprehensive *T. molitor* immune gene expression information at the transcriptional level following parasitization, and sheds valuable light on the molecular understanding of the host-parasitoid interaction.

## Introduction

Multicellular animals are continually exposed to foreign invaders such as viruses, bacteria, fungi, protozoans as well as various metazoan parasites and parasitoids. In order to defend themselves against invaders, they have evolved two effective immune systems known as acquired and innate immunity. The acquired immune system relies on the generation of random and highly diverse repertoires of antigens that allow organisms to develop an immunological memory, whereas the innate immune system uses germline encoded factors to recognize and kill foreign invaders [1], [2]. Vertebrates possess both acquired and innate immunity. Insects and other invertebrates lack the acquired immune system but have a well-developed innate immune system that successfully combats foreign invaders. The first defense of insects against invaders is the physical barriers, including the cuticle and peritrophic matrix [3]. Once the invaders overcome the physical barriers, insects will provoke a complex series of immune reactions to cope with the invaders. The immune system of insects can be divided into two categories: (i) humoral defense, including the antimicrobial peptides, reactive intermediates of oxygen or nitrogen, melanin formation and clotting; and (ii) cellular defense mainly based on haemocytes, such as phagocytosis, encapsulation, microaggregation and nodulation [4]–[6].

Parasitoids are insects that spend a significant portion of their life in the body or on the body surface of other invertebrates, mostly other insects, and whose larvae feed on and eventually kill the host [7]. During this process, oviposited eggs and developing larvae must contend with host immune responses. An amazing array of mechanisms have been evolved that enable parasitoids to effectively evade or deactivate host defenses [8]. These include introduce or secrete various virulent factors such as venom, polydnaviruses and virus like particles into host's body upon parasitization [9]–[11]. Most studies have focused on these parasitic factors and their effects on the host. Despite both their molecular identity and physiological role involving in hosts immune suppression have been investigated to various extents in many parasitoid-host systems, the transcriptional immune responses of host to parasitic wasps are still poorly understood [12]–[15]. This research trajectory has only over the last several years become a focal point of research. In addition, most of these researchers so far have only focused on the differential expression of individual or small group of host genes following parasitization. The recent development of transcriptome and digital gene expression (DGE) based on next generation deep-sequencing technology provides extensive data in much shorter time period with enormous depth and coverage to globally analyse the blueprint of host's gene expression profile under parasitization challenge.

The ant-like bethylid wasp *Scleroderma guani* (Hymenoptera: Bethylidae) is a generalist ectoparasitoid of wood-boring insects, which is widely dispread in China and has been firstly discovered in Guangdong and Shandong provinces in 1973 and 1975, respectively [16], [17]. In China, since its discovery, *S. guani* as a biocontrol agent has been widely used to control longhorned beetles, including *Monochamus alternatus* that is the most important insect vector of the pinewood nematode *Bursaphelenchus xylophilus*, the causal agent of pine wilt disease [18]. This polyphagous parasitoid can parasitize larval or pupal stage of more than 50 species of insects belonging to 22 families from three orders, including the yellow mealworm beetle, *Tenebrio molitor* (Coleoptera: Tenebrionidae) [19]. *T. molitor* pupa is one of the preferred hosts for mass rearing *S. guani* in the laboratory.

For the present study, we used the Illumina sequencing to explore the *T. molitor* immune response induced by *S. guani* parasitization. Firstly, we obtained and characterized the transcriptome of *T. molitor* pupae with special emphasis on immune related genes. Additionally, we constructed two DGE libraries and compared the gene expression profiles of non-parasitized and parasitized *T. molitor* pupae. A combination of transcriptome and DGE analyses revealed key differentially expressed immune genes in parasitized versus non-parasitized *T. molitor* pupae. The results give us a comprehensive view of global gene expression profiles of host response to parasitization, and shed light on the molecular basis of host-parasitoid interaction.

## Materials and Methods

### Ethics statement

Regarding the field study, no specific permits were required. The location is not privately-owned or protected in any way. The field studies did not involve endangered or protected species.

### Insects and parasitization

*T. molitor* were mass reared under natural photo regime and temperature conditions on wheat bran. Vegetables were placed on top of the bran to provide water. *S. guani* strain was originally collected from the pine forestry in suburb of Kunming, China. The parasitoid had been maintained in the laboratory for over 2 years on *T. molitor* pupae. After emergence, adult parasitoids were fed with 20% honey solution. Parasitization by *S. guani* was carried out by exposing newly pupated pupae of *T. molitor* to female wasps. When a pupa was seen to be parasitized, it was transferred to a fresh glass petri dish, where it was allowed to develop at 25°C before harvesting for RNA extraction.

### cDNA library preparation and Illumina sequencing

Total RNA was isolated from the pupae at various developmental stages separately using TRIzol® Reagent (Invitrogen) according to the manufacturer's instructions. Then, all total RNAs were pooled together. RNA integrity was confirmed using the 2100 Bioanalyzer (Agilent Technologies). The mRNA was enriched from 20 μg total RNA using oligo(dT) magnetic beads and fragmented with RNA Fragmentation Reagent. The cleaved RNA fragments were transcribed into the first-strand cDNA using random hexamer-primer, followed by second-strand cDNA synthesis. Short fragments were then purified with a QiaQuick PCR extraction kit (Qiagen, Valencia, CA, USA) and were resolved with EB buffer for end reparation and adding poly(A). After that, they were ligated to sequencing adapters. Finally, these products were purified and enriched with PCR to create the final cDNA library. The library was sequenced using Illumina HiSeq™ 2000 platform. Raw data were deposited to DDBJ database under accession DRA000603.

### Transcriptome analysis

Data analysis and base calling were performed by the Illumina instrument software. After removing the sequence reads containing sequencing adapters and low quality sequence reads, the clean reads were assembled using SOAPdenovo software [20]. The resultant contigs were joined into scaffolds using paired-end joining and gap-filling. Paired-end reads are used again for gap filling of scaffolds to get sequences with least Ns and cannot be extended on either end. The final sequences were named unigenes. For assignments of predicted gene descriptions, the assembled unigenes were compared to the protein of NCBI nr databases with the BLASTX algorithm at the threshold of E-value <10^−5^. For functional annotation, the Gene Ontology (GO), Cluster of Orthologous Groups (COG), and Kyoto Encyclopedia of Genes and Genome (KEGG) annotation for each unigene was performed using the automatic annotation tool Blast2GO program [21].

### DGE library preparation and sequencing

Total RNA was extracted separately from non-parasitized and parasitized *T. molitor* pupae at 6, 12, 24 and 48 h post-parasitization following the manufacturer's instruction, as described above. Total RNAs extracted from the two different treatments were pooled together. Next, a DGE library was prepared using an Illumina gene expression sample prep kit. Briefly, mRNA was enriched by using the oligo(dT) magnetic beads from the total RNA, and cDNAs were synthesized as described above for each sample. Bead-bound cDNA was digested with the restriction enzyme Nla III, which recognizes and cuts off the CATG sites. Those cDNA fragments with 3′ ends were purified from the magnetic beads, and Illumina adaptor 1 was added to their 5′ ends. After digestion with Mme I, which recognizes the junction of the adapter 1 and the CATG site, and makes a cut at 17 bp downstream of the NlaIII recognition site, 21-bp tags containing adaptor 2 were ligated to the 3′ ends of the tags to create a tag library. The required fragments were enriched by linear PCR amplification for 15 cycles and purified by 6% TBE PAGE gel. After denaturation, the single-chain molecules were anchored to Illumina chip, and sequenced via Illumina HiSeq™ 2000.

### DGE analysis

The original image data was transferred into sequence data by base calling. The raw data were filtered to get the clean reads by removing the dirty reads with adaptors, low-quality reads (reads with unknown sequences), empty reads (no read sequence between the adaptors), and reads with only one copy number (probable sequencing error). Then, clean reads were mapped to the above transcriptome reference database using SOAPaligner/soap2 [22], allowing no more than 2 mismatches. The remainder of the clean tags were designed unambiguous clean reads. The unigene expression level was calculated by using RPKM (reads per kb per million reads) method [23]. To identify the DGEs in the two samples, a strict algorithm was developed for statistical analysis according to the method of Audic and Claverie [24]. False discovery rate (FDR) is a method to determine the threshold of P-value in multiple tests. During the analysis, “FDR ≤0.001 and the absolute value of log2Ratio ≥1” was used as the threshold to judge the significance of gene expression difference. Next, all DGEs were subjected to GO and KEGG enrichment analysis compared to the transcriptome background using hypergeometric test. The calculated P-value goes through Bonferroni Correction, taking corrected P-value ≤0.05 as a threshold. GO terms or KEGG pathways fulfilling this condition were defined as significantly enriched terms in DEGs.

### Quantitative real-time PCR (qRT-PCR) validation

Total RNA was extracted as described for DGE library preparation and sequencing, and then was reverse-transcribed according to the protocol provided with the M-MLV first strand kit (Invitrogen, China). Thirteen differentially expressed genes were randomly selected to verify DGE sequencing results using three replicates. The actin gene was used as a constitutive expression control for normalization. The primers are shown in Table S1. qRT-PCR was carried out using a BIO-Rad IQ5 Real-Time PCR system (Bio-Rad) under the following conditions: 95°C for 5 min; and 40 cycles of 95°C for 10 s, 60°C for 15 s, and 72°C for 20 s, followed by melting curve generation (68°C to 95°C). EvaGreen (Bio-RAD, U.S.) was used as DNA-binding fluorescent dye according to the manufacturer's protocol.

## Results and Discussion

### Illumina sequencing and assembly

**Figure 1:**
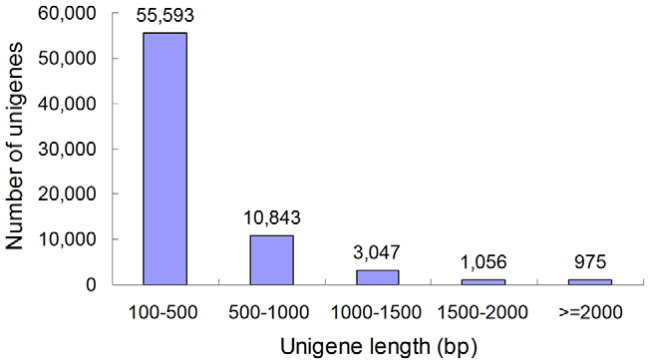
Length distribution of *Tenebrio molitor* unigenes. Horizontal axis represents the length of unigenes and vertical axis represents number of unigenes.

A total of 264,698 raw reads were generated through one pyrosequencing run (Table 1). An average read length was 90 bp, which is consistent with the Illumina sequencing capacity. The GC percentage of the reads is 44.03%, which is comparable with genome sequence of other insects. After cleaning of dirty reads and quality checks, these short reads were assembled into 389,298 contigs (Figure S1). These contigs were further assembled into 106,202 scaffolds with a mean length of 325 bp by using paired end-joining and gap-filling (Figure S2). Clustering of these scaffolds revealed 71,514 unigenes. They ranged from as small as 150 bp to as large as 12518 bp. The mean size of them was 424 bp. The lengths of the 5,078 unigenes were more than 1,000 bp (Figure 1). Most unigenes were shorter than 500 bp, and only near 25% of them were longer than 500 bp, which is similar to that obtained in previous transcriptome analyses in different insects using Illumina platform [25], [26]. This drawback might be resulted from the short length of the sequencing read. In addition, successful assembly of next generation sequencing data into relatively long sequences is affected by the coverage of individual reads [27]. The unigene could be lengthened by the increased sequence depth using normalized library with 454 platform.

**Table 1:** Sequence statistics of the Illumina sequencing assembly.

	Reads	Contigs	Scaffolds	Unigenes
Number of sequences	26,666,668	389,298	106,202	715,14
Mean length (bp)	90	148	325	424
Total length (bp)	2,400,000,120	57,799,627	34,470,701	30,319,407

### Functional annotation and classification

**Figure 2:**
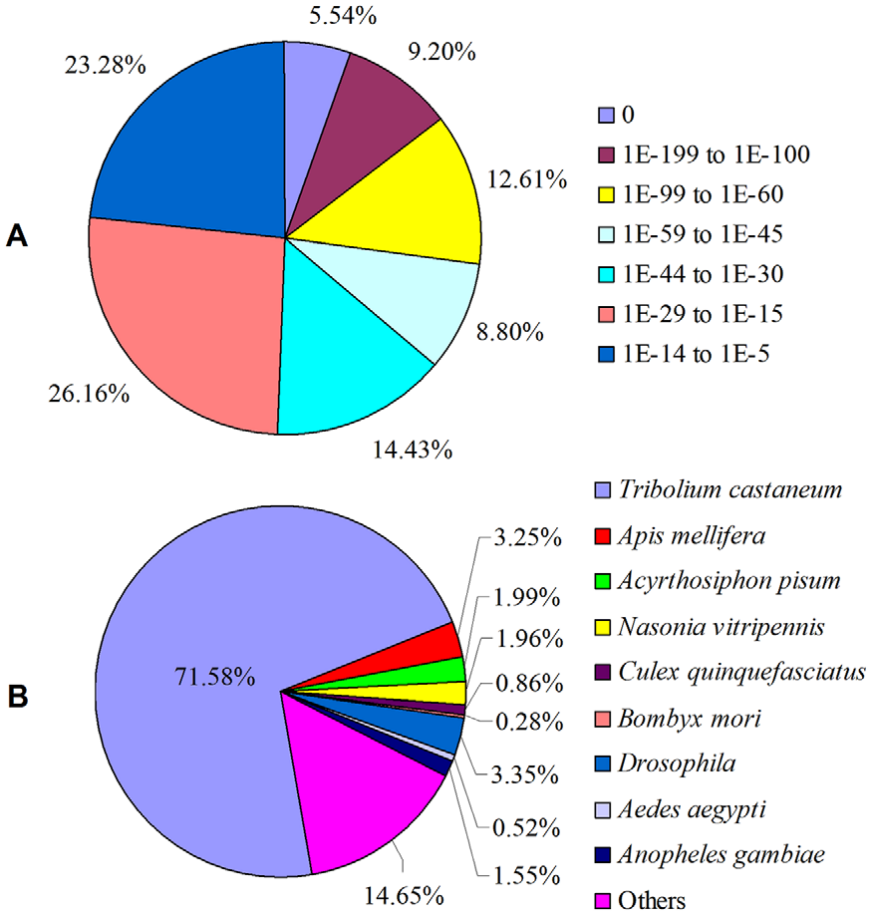
Homology analysis of *Tenebrio molitor* unigenes. (A) E-value distribution. (B) Species distribution. All unigenes that had BLASTX annotations within the NCBI nr database with a cut-off E-value of 10^−5^ were analyzed. We used the first hit of each sequence for analysis.

For BLASTX annotation, unigenes were searched against proteins of the NCBI nr database using a cut-off E-value of 10^−5^, just over half (52.26%) of all unigenes returned an above cut-off BLAST result. The remaining 34,141 unigenes (48.74%) could not be done and need more genetic data to annotate. As longer sequences were more likely to obtain BLAST matches in the protein databases [28], the relatively low annotated percentage is partially resulted by a high frequency of short sequences in our database. This also might be due to the transcripts derived from the cDNA of untranslated regions, chimerical sequences (assemblage errors) and nonconserved areas of proteins where homology is not detected, which could be resolved by enhancing the accuracy of the assembly and perfecting gene annotation strategies [29], [30]. Additionally, it could be estimated that a large part of the genes in *T. molitor* transcriptome database are with unknown functions. As shown in Figure 2A, among the annotated unigenes, 13,507 (36.15%) showed strong homology (smaller than 1.0E-45) (Table S2), whereas 8,699 (23.28%) showed poor matches with E-values between 1E-14 and 1E-5. For species distribution, most (71.58%) of the BLASTX hits are matched to the *Tribolium castaneum* sequences followed by *Drosophila* (3.35%), *Apis mellifera* (3,25%) and *Acyrthosiphon pisum* (1.99%) (Figure 2B). It is in accordance with the results of transcriptome profiling of other beetles including *Tomicus yunnanensis* and *Dendroctonus ponderosae*, of which over 60% genes were most closely related to *T. castaneum* [26], [30], [31]. This can be explained by the fact that *T. castaneum* genome is the only fully sequenced beetle genome and currently represents the vast majority of deposited coleopteran sequences in NCBI [32].

**Figure 3:**
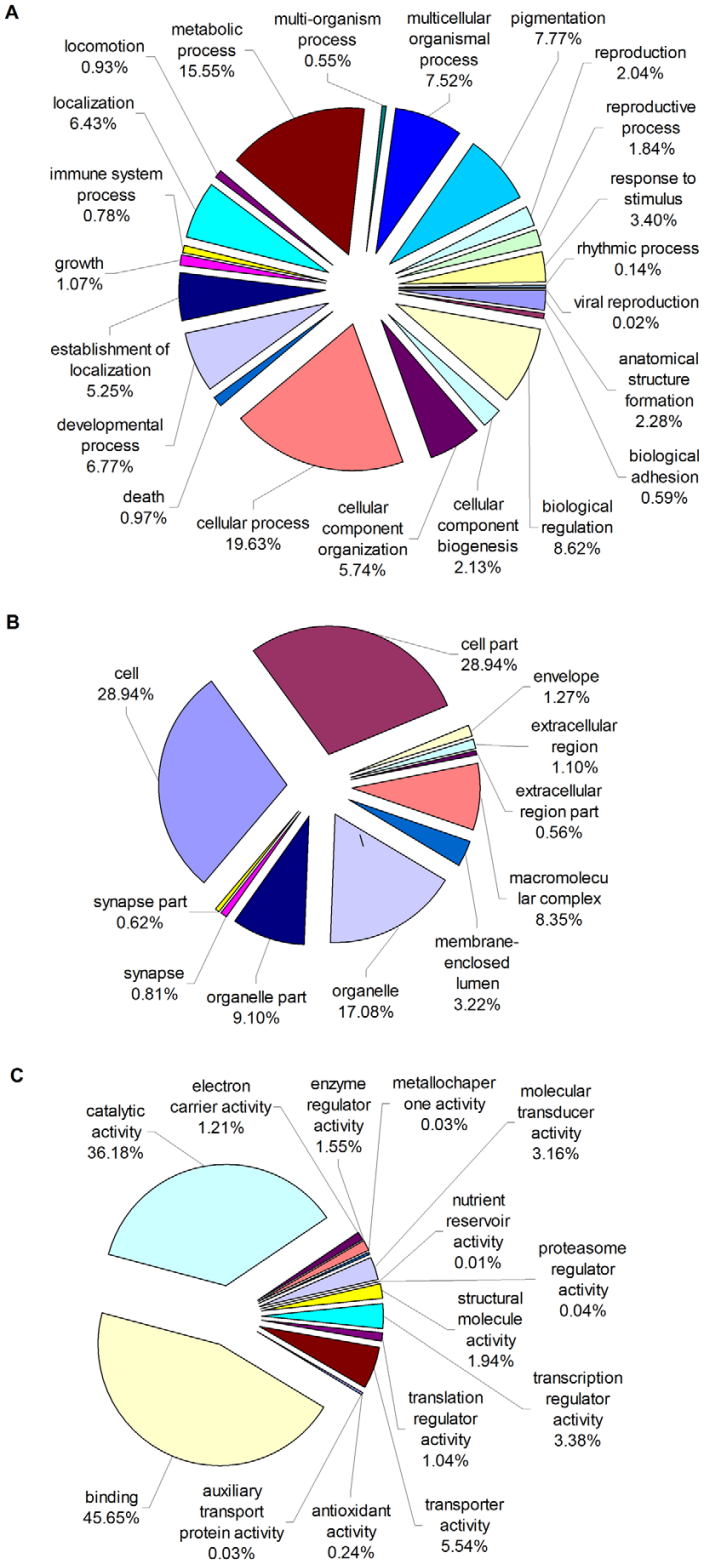
Gene ontology (GO) classification of *Tenebrio molitor* unigenes. Categories for (A) biological processes, (B) cellular components, and (C) molecular processes. The data presented represent the level 2 analysis. The percentage of a specific category of genes in that main category is shown.

Based on the ‘best hit’ BLASTX search, unigenes were further assigned to GO classification using Blast2GO. A total of 51,301 unigenes were annotated and assigned to GO terms (Table S3). They were classified into the three main GO categories: biological process, cellular component, and molecular function, covering with a wide range of subcategories. 23,831 (46.45%) unigenes were grouped under biological process, 17,179 (33.49%) under cellular component, and 10,291 (20.06%) under molecular function (Figure 3). Under the category of biological process, cellular process (19.63%) and metabolic process (15.55%) represented the most abundant subcategories, indicating the importance of cell cycle, generation as well as metabolic activities in this developmental stage. In the category of cellular component, cell (28.94%) and cell part (28.94%) represented the most abundant subcategories followed by organelle (17.08%) and organelle part (9.10%). The molecular function category was mostly dominated by the group of binding (45.65%) and catalytic activity (36.18%). With regard to the subcategories that took up a large proportion, similar observations for them were reported in transcriptomic studies of other insects [29], [33]. Comparing *T. molitor* with *T. yunnanensis* and *D. ponderosae* in the transcriptome-wide GO classification [26], [30], [31], they had high similarity with each other in the percentages of subcategory assignations for the three main categories. *T. molitor* sequences fall into GO categories with a roughly similar distribution to that of *T. castaneum* genome. It suggests that the sequencing provided a comprehensive representation of the *T. molitor* transcriptome, and the *T. molitor* sequence data do not contain notable biases towards particular categories of genes.

**Figure 4:**
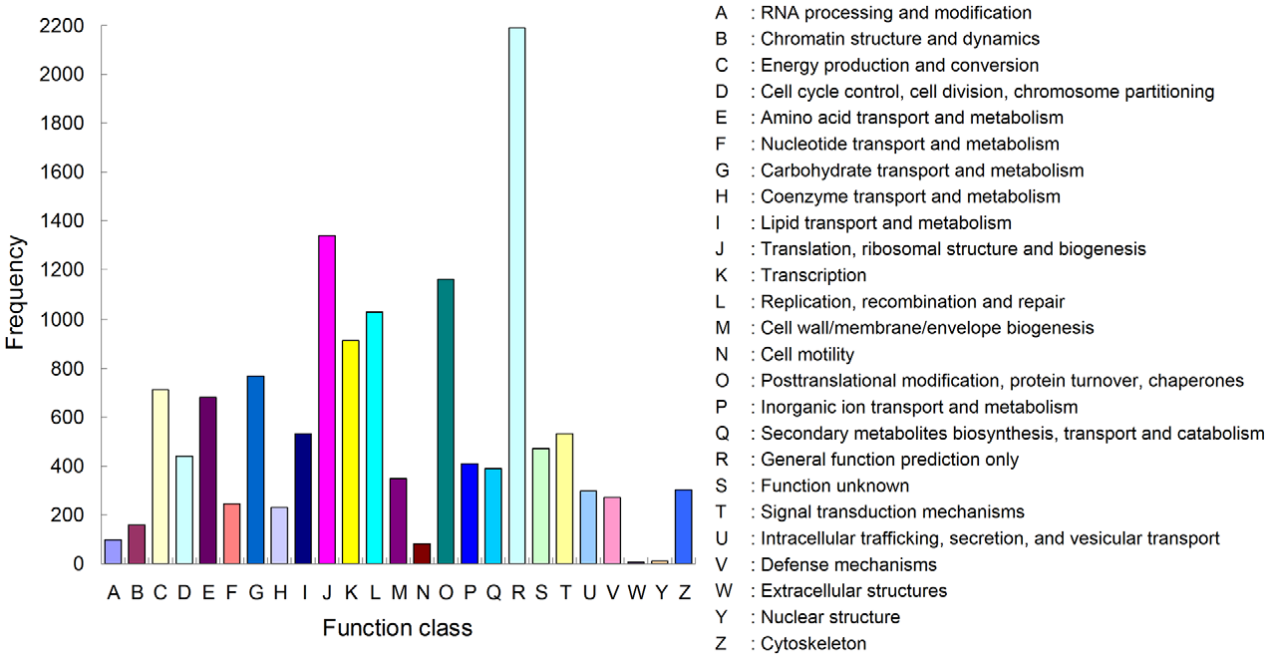
Clusters of orthologous groups (COG) classification of *Tenebrio molitor* unigenes. A total of 13,599 produced functional annotations were among the 25 categories.

To further analyze the putative protein functions, the assembled unigenes were compared against COG. Altogether 13,599 functional annotations were produced, which were assigned to the appropriate COG clusters. These COG classifications were grouped into 25 functional categories (Figure 4). The largest category was general function prediction only, which contained 2,189 unigenes (16.10%). It was followed by translation, ribosomal structure and biogenesis (1,340, 9.85%), posttranslational modification, protein turnover, chaperones (1,162, 8.54%), and replication, recombination and repair (1,026, 7.54%). Only 3 unigenes (0.02%) belonged to extracellular structures, which was the smallest group. Then, nuclear structure, cell motility, and RNA processing and modification were the relatively smaller groups, containing 12, 83 and 95 unigenes, respectively.

More specifically, KEGG pathway analysis was performed on unigenes to identify the biological pathways active in *T. molitor*. As a result, there were 21,022 unigenes assigned to 205 KEGG pathways (Table S4). Highest number of sequences belonged to metabolic pathways (3,440, 16.36%) followed by spliceosome, pathways in cancer, purine metabolism, regulation of actin cytoskeleton, endocytosis, focal adhesion, Huntington's disease, tight junction, lysosome and so on. Except for metabolic pathways, the gene numbers and percentages of other pathways were below 1,000 and 5%, respectively.

### Putative immune related genes

Well-categorized and annotated transcriptome serve as important and valuable resources for gene identification. Despite 50 of the 284 *T. molitor* proteins including serine proteinase (SP) and its homologue (SPH), serpin, β-1,3-glucan recognition protein (GRP)/ gram-negative binding protein (GNBP), peptidoglycan recognition protein (PGRP), prophenoloxidase (PPO), and melanization inhibitor that participate in immune responses were available in the NCBI database (prior to July 22, 2012), these molecules appear to represent only a small portion of the *T. molitor* immune system. In order to gain deep insight into the molecular biology of immune systems in *T. molitor*, the immune-relevant genes were discovered by referencing to those identified in *T. castaneum* genome [34]. Table 2 gives an overview of immune-related unigenes identified. 430 unigenes were noticed with significant similarity to a variety of immune system-related genes, revealing the presence of an elevated number of relevant molecules for the immune response (Table S5). This finding further demonstrates the high quality of our sequencing data. Based on molecular functions, immunity genes in insects can be assigned to three groups: effector genes encoding proteins that directly inhibit pathogen growth and survival, recognition genes that encode pathogen surveillance proteins, and signaling genes that encode proteins involved in immune-related signaling pathways [35]. Among the discovered sequences, the transcripts involved in these three different functional groups such as effector genes (e.g., antimicrobial peptide (AMP), and nimrod), recognition genes (e.g., PGRP, GRP, scavenger receptor (SR), and thiolester containing protein (TCP)), and signaling genes (e.g., Toll, relish and tab2) were identified. In *T. castaneum* genome, 388 immune genes have been identified [34], which are lower than those of *Bombyx mori* (203), *D. melanogaster* (268) or *Anopheles gambiae* (297) but are substantially higher than those of *A. mellifera* (121) [36]. Although the current number of immune sequences in *T. molitor* is more than that obtained from fully sequenced *T. castaneum* genome, it is noteworthy that some remainders might be absent. There was evidence of existence of few immune gene families such as spätzle, glutathione oxidase, and heme peroxidase that were not present in our transcriptome database. Furthermore, many unigenes obtained by next generation sequencing should be different fragments or even allelic or splice variants of the same gene [37]. In this study, the number of gene sequences predicted to encode immune genes would be therefore an overestimate of the actual number of genes belonging to each of the currently characterized gene families. The putative genes uncovered provide the basis for further identification of the physiological functions of candidate genes in *T. molitor* immune responses. However, most of these interesting genes are partial sequences. Their full-length sequences are needed to be obtained by RACE PCR for further study.

**Table 2:** Number of immunity-related genes in *Tenebrio molitor* transcriptome and *Tribolium castaneum* genome.

Gene name	Gene numbers	
	*Tenebrio molitor* a	*Tribolium castaneum* b
Peptidoglycan recognition protein	15	6
β-1,3-Glucan recognition protein/Gram-negative binding proteins	9	3
C-type lectin	24	16
Galectin	6	3
Fibrinogen-like	4	7
Thioester-containing protein	3	4
Serine proteinase or its homolog	90	168
Serpin	87	35
Spätzle	0	7
Toll-like receptor	2	9
MD2-like	0	8
Cactus	2	1
Pelle	1	1
Myd88	3	1
Tube	1	1
Pellino	3	1
Traf	0	1
Cactin	7	1
Relish	6	4
FADD	0	1
IKKb	0	2
IKKg	0	1
IMD	0	1
TAK	0	1
Caspase	15	9
IAP	0	4
Tab2	2	1
Hep	0	1
Basket	3	3
Jra	0	1
Kay	0	1
DOME	0	1
HOP	6	1
STAT	0	1
Prophenoloxidase	12	3
Melanization inhibitor	0	1
Hexamerin	23	6
Catalase	7	4
Glutathione oxidase	0	3
Heme peroxidase	0	11
Peroxiredoxin	10	6
Superoxide dismutase	8	4
Antimicrobial peptide	14	17
Neuroglian/hemolin	7	1
Scavenger receptor	36	21
Nimrod	13	4
Draper	11	1

a Number of sequences obtained in this study that annotated with NCBI nr database.

b Number of sequences from Zou et al. (2007).

### DGE library sequencing

Using DGE method, the gene expression profile of *T. molitor* in response to parasitization by *S. guani* was globally analyzed. Two DGE libraries were sequenced from the pooled non-parasitized and parasitized pupae at four different time intervals post parasitization using parallel sequencing on the Illumina platform. A total of 6.07 and 6.16 million raw reads were generated, respectively (Table 3). After removing the low quality reads, 6,005,103 clean reads for the non-parasitized pupae and 6,102,041 clean reads for the parasitized pupae were obtained. About 99% of the raw reads passed the filter, reflecting the high quality of sequencing (Figure S3). The number of read entities with unique nucleotide sequences was 52,038 for non-parasitized pupae and 53416 for parasitized pupae, which was similar between each library. In addition, the distribution of the read expression over different read abundance categories demonstrated that both libraries displayed a similar pattern (Figure S4). These indicate that there was no bias in the construction of the libraries and the DGE data had good normality. Among the distinct clean reads, around 17% of them were high-expression reads with copy numbers larger than 100, with around 32% appearing below 6 times and most of them (around 50%) appearing between 6 and 100 times.

**Table 3:** Statistics of DGE sequencing.

	NP	P
Raw data	6,072,324	6,164,853
Total clean reads	6,005,103	6,102,041
Total basepairs	300,255,150	305,102,050
Total mapped	3,023,449	2,968,527
Perfect match	2,267,131	2,226,161
< = 2 bp mismatch	756,318	742,366
Unique match	3,022,715	2,967,863
Multi-position match	734	664
Total unmapped	2,981,654	3,133,514

NP, non-parasitization. P, parasitization.

After mapping to the above created transcriptome database, 3,023,449 and 2,968,527 total mapped reads were acquired for non-parasitized and parasitized pupae, respectively (Table 3). Of the total clean reads, 50.34% and 48.64% of distinct clean reads uniquely matched to unigenes, only 0.01% mapped to multiple unigenes, and 49.65% and 51.35% did not map to the dataset. The lower mapped ratio might result from insufficient reference transcriptome data. It also would be due to the incomplete NlaIII digestion during library preparation, many reads matching noncoding RNAs and the usage of alternative polyadenylation and/or splicing sites [38]. Additionally, incomplete *T. molitor* genome sequencing is the most likely reason. In order to further evaluate the unique mapping reads, we analyzed the distribution of gene coverage in each sample, which is the number of distinct clean reads that aligned to the reference genes (Figure S5). Very similar coverage patterns were observed in each library. Most gene coverage was >50% in each sample, with approximate 28% appearing between 90–100%. Fewer than 2% of the genes were covered by 0–10%.

### Identification of DGEs

**Figure 5:**
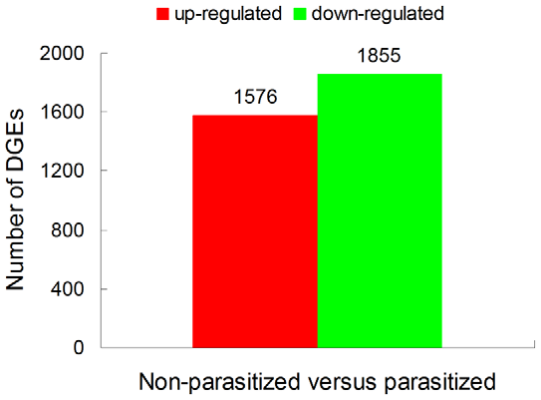
Changes in gene expression profile between non-parasitized (NP) and parasitized (P) *Tenebrio molitor* pupae. The number of up-regulated and down-regulated genes between NP and P groups is summarized here.

To gain insight into the global transcriptional changes taking place in *S. guani* parasitized *T. molitor* pupae, the DGEs were identified from the normalized DGE data by pairwise comparisons between non-parasitized and parasitized libraries. After parasitization, 3,431 mapped genes showed significant changes in expression. The up-regulated and down-regulated genes were shown in Figure 5. The number of down-regulated genes was larger than that of up-regulated. The detected fold changes (log2 ratio) of gene expression ranged from −16.48 to 17.96 (Table S6). 94.80% and 97.68% of genes in the two DGE libraries were up- or down-regulated between 1.0- and 5.0-fold. To investigate the most differentially up-and down-regulated genes, top 20 up-regulated and 20 down-regulated genes were screened out (Table S7). Only 20 genes had hits in the NCBI nr database, such as cathepsin B10 cysteine protease, reverse transcriptase-like protein, fatty acid synthase S-acetyltransferase, and cytochrome P450. Interestingly, it has been reported previously that cytochrome P450 known to be insecticide resistance/detoxification was up-regulated following parasitism, while it was the top down-regulated genes after parasitization in this study [39]. In *Plutella xylostella* larvae parasitized by *Diadegma semiclausum*, the transcripts of two cytochrome P450 genes were changed with one up-regulated and another down-regulated [40]. With respect to half of the top changed genes with no defined functions, it is consistent with the result that near 50% unigenes in the above transcriptome database remains to be annotated. However, it indicates that DGE tool has contributed greatly to identify novel genes involved in parasitoid and host interaction.

**Figure 6:**
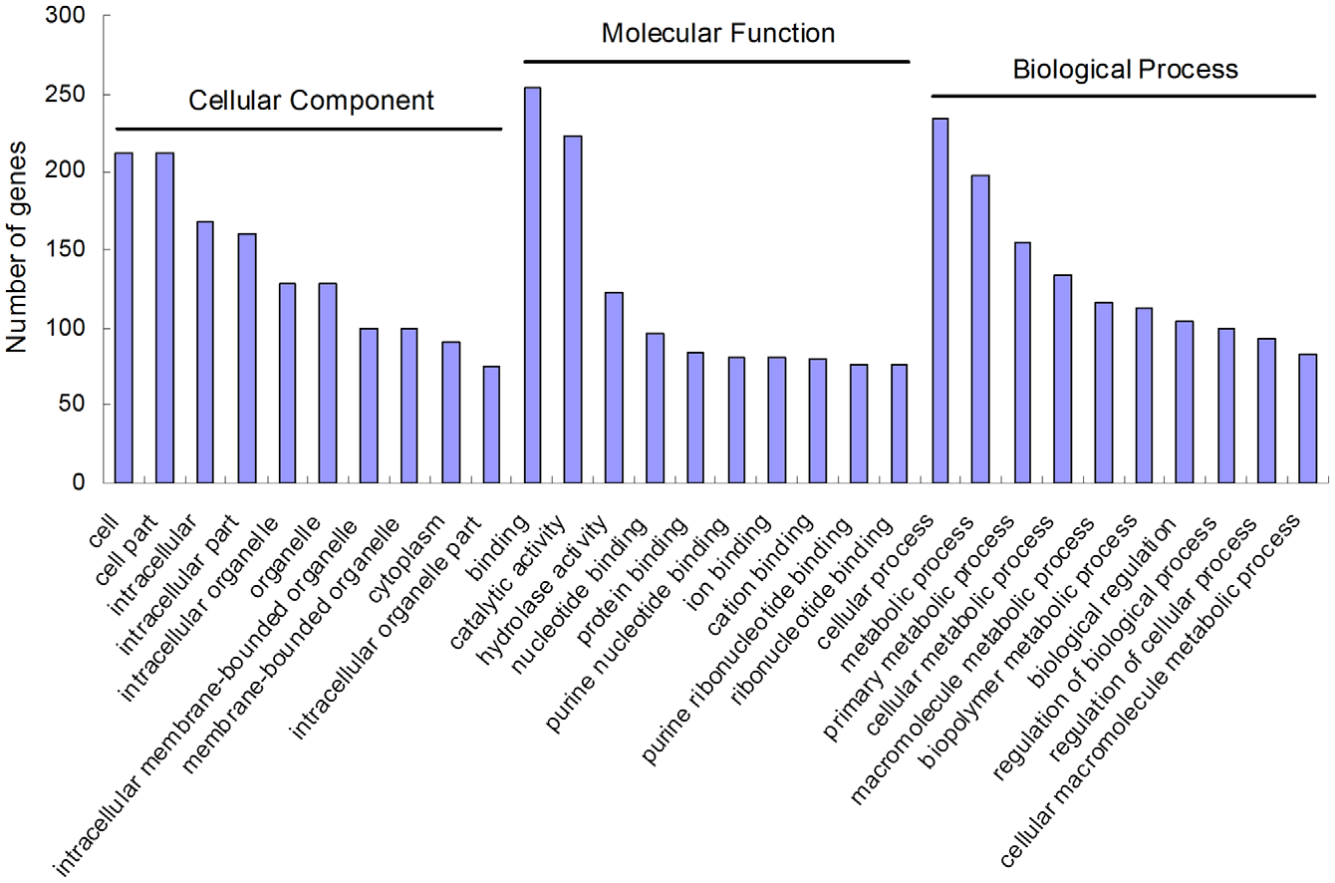
Selected major enriched GO terms. Transcripts were annotated in three categories: cellular components, molecular function, and biological processes.

To facilitate the global analysis of gene expression, GO and KEGG enrichment analysis were performed by mapping each differentially expressed gene into the records of the GO and KEGG database. Of the enriched GO categories, the major subcategories were as follows: cell and cell part in the cellular component cluster; binding and catalytic activity in the molecular function cluster; and cellular process and metabolic process in the biological process cluster (Figure 6). There were 9, 15 and 14 GO terms significantly enriched in the cellular component, molecular function and biological process, respectively (Table S8). For the KEGG analysis, the major enrichment was observed in metabolic pathways, purine metabolism, Huntington's disease, vibrio cholerae infection, and MAPK signaling pathway (Table S9). Thirty five pathways were significantly enriched. Interestingly, MAPK signaling pathway, metabolic pathways and insect hormone biosynthesis were among them.

**Figure 7:**
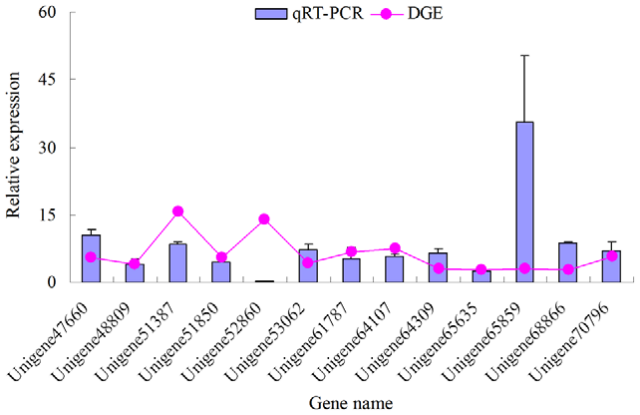
Verification of differentially expressed genes by qRT-PCR. The relative expression levels of these unigenes indicate the log2 ratio of non-parasitized (NP) and parasitized (P) *Tenebrio molitor* pupae by DGE and qRT-PCR, respectively. Error bars indicate standard deviations of averages from three replicates.

To validate the DGE result, qRT-PCR analysis was performed using gene-specific primers for the 13 up-regulated unigenes selected at random. The results showed that all unigenes exhibited the up-regulated expression trend in qRT-PCR analysis as the original DGE results (Figure 7). This illustrates that the results of gene expression profiling from DGE are reliable. It is worth noting that the expression levels of Unigene51387 and Unigene65859 were obviously different between qRT-PCR and DGE. This difference might be caused by a sensitivity of biases occurred between qRT-PCR and DGE.

### DGEs involved in immune response

By combining analysis of the indentified putative immune genes and DGEs, 16 out of the 47 immune gene families containing 74 unigenes were found to be respondent to parasitization (Table 4). They included AMP, C-type lectin (CTL), PGRP, GRP/GNBP, PPO, SP, serpin, superoxide dismutase (SOD), peroxiredoxin, hexamerin, cactus, pellino, cactin, caspase, and catalase. The highest changed expression levels in these genes were 6.77 fold for AMP and −13.12 fold for peroxiredoxin. There were 52 unigenes altered lower than two-fold change. As the transcriptional levels of some genes might be distinctly changed at early or late stage, the low fold change would be resulted by the pooled samples from different time intervals ranged from 6–48 h post-parasitization. Also, other immune genes whose transcription levels related to parasitization by *S. guani* could be revealed by detailed analysis of the samples from each time point.

**Table 4:** A list of *Tenebrio molitor* immune-related genes that were differentially transcribed after parasitization by *Scleroderma guani*.

GeneID	Length	NP-RPKM	P-RPKM	Fold change	P-value	FDR
**Antimicrobial peptide**						
Unigene48479	283	24.55	183.35	2.90	2.03E-09	5.64E-08
Unigene8990	394	20.99	141.96	2.76	1.63E-10	5.32E-09
Unigene12213	416	34.20	140.93	2.04	1.14E-13	6.38E-12
Unigene15283	392	56.54	312.02	2.46	0	0
Unigene22242	159	76.99	504.35	2.71	2.13E-13	1.10E-11
Unigene61787	473	5.60	610.49	6.77	5.63E-06	8.58E-05
**Lysozyme**						
Unigene43694	251	358.51	122.16	−1.55	1.59E-21	1.76E-19
Unigene46654	269	290.24	71.40	−2.02	1.56E-26	2.14E-24
**C-type lectin**						
Unigene11733	1225	85.34	34.38	−1.31	1.49E-19	1.48E-17
Unigene16572	921	4.31	15.00	1.80	3.98E-05	0.00048907
Unigene25850	172	94.25	31.34	−1.59	4.57E-05	0.00055274
Unigene64817	578	11.45	34.98	1.61	3.85E-06	6.04E-05
Unigene69689	1051	7.55	26.61	1.82	3.44E-09	9.27E-08
**Peptidoglycan recognition protein**						
Unigene2556	689	10.56	40.10	1.92	2.00E-09	5.56E-08
Unigene46415	268	60.49	218.76	1.85	3.82E-14	2.44E-12
Unigene54494	341	74.70	761.83	3.35	0	0
Unigene65399	605	70.54	226.67	1.68	0	0
Unigene68368	834	12.30	28.28	1.20	6.35E-05	0.00073674
**β-1,3-Glucan recognition protein/ Gram-negative binding proteins**						
Unigene59692	425	78.62	233.88	1.57	0	0
Unigene69724	1058	49.72	128.34	1.37	5.28E-14	3.24E-12
**Prophenoloxidase**						
Unigene20627	154	406.02	109.40	−1.89	7.58E-20	7.64E-18
Unigene20889	155	192.09	58.69	−1.71	4.84E-09	1.27E-07
Unigene37907	221	667.64	167.71	−1.99	8.13E-48	1.77E-45
Unigene40274	232	1232.05	325.32	−1.92	9.24E-87	2.38E-84
Unigene68337	830	536.90	135.99	−1.98	9.24E-140	2.49E-137
Unigene11469	539	25.17	6.88	−1.87	3.00E-05	0.00038056
**Serine protein or its homolog**						
Unigene1207	1292	10.75	29.21	1.44	5.38E-09	1.40E-07
Unigene13777	892	14.46	32.49	1.17	1.44E-05	0.000199
Unigene2204	386	42.00	13.97	−1.59	4.57E-05	0.000553
Unigene2906	208	17.50	79.38	2.18	1.30E-06	2.25E-05
Unigene39230	227	148.65	400.77	1.43	0	0
Unigene41772	240	64.79	190.93	1.56	9.09E-12	3.45E-10
Unigene45616	263	42.77	98.65	1.21	2.57E-05	0.000332
Unigene51777	311	61.70	222.10	1.85	7.77E-15	5.51E-13
Unigene54421	340	12.65	74.33	2.55	2.93E-07	5.75E-06
Unigene60508	442	62.87	190.58	1.60	0	0
Unigene61013	454	160.31	414.87	1.37	3.00E-13	1.49E-11
Unigene61795	474	25.82	105.92	2.04	2.13E-13	1.10E-11
Unigene69393	989	122.76	58.26	−1.08	8.18E-17	6.86E-15
Unigene69732	1058	44.40	140.45	1.66	2.60E-13	1.30E-11
Unigene70037	1131	551.38	1828.31	1.73	2.26E-12	9.35E-11
Unigene9960	952	34.75	2.83	−3.62	2.96E-21	3.24E-19
**Serpin**						
Unigene1138	230	1329.07	438.03	−1.60	2.76E-72	6.94E-70
Unigene1484	775	10.67	54.35	2.35	1.63E-10	5.32E-09
Unigene16750	547	99.79	38.81	−1.36	1.80E-11	6.58E-10
Unigene34207	204	77.84	181.68	1.22	3.50E-07	6.78E-06
Unigene44272	254	61.22	14.59	−2.07	1.75E-06	2.95E-05
Unigene45167	260	27.99	116.63	2.06	1.10E-09	3.18E-08
Unigene54405	339	105.40	44.73	−1.24	4.73E-07	8.96E-06
Unigene55316	351	67.86	233.27	1.78	0	0
Unigene57316	381	26.92	305.99	3.51	3.98E-12	1.58E-10
Unigene59916	429	45.50	223.06	2.29	1.22E-14	8.38E-13
Unigene60952	453	70.84	238.02	1.75	4.69E-14	2.91E-12
Unigene60995	454	67.77	138.04	1.03	8.35E-09	2.11E-07
Unigene63284	518	68.98	26.02	−1.41	2.66E-08	6.26E-07
Unigene69118	940	82.36	333.36	2.02	4.38E-13	2.07E-11
Unigene69212	958	67.69	17.59	−1.94	3.11E-21	3.39E-19
Unigene69220	959	63.82	460.97	2.85	0	0
Unigene70002	1119	68.00	29.51	−1.20	5.38E-13	2.47E-11
**Superoxide dismutase**						
Unigene66938	701	11.80	1.44	−3.03	1.88E-05	0.00025097
**Peroxiredoxin**						
Unigene14679	593	8.93	0.00	−13.12	1.78E-05	0.00023959
Unigene40514	233	160.44	368.76	1.20	2.62E-13	1.31E-11
**Hexamerin**						
Unigene16646	1348	138.42	297.95	1.11	8.62E-13	3.82E-11
Unigene18014	150	760.91	276.29	−1.46	1.03E-24	1.33E-22
Unigene28282	181	1398.25	571.50	−1.29	5.26E-44	1.08E-41
Unigene30910	191	1032.32	365.17	−1.50	4.65E-43	9.28E-41
Unigene36412	214	1290.85	603.03	−1.10	1.98E-37	3.62E-35
Unigene65901	631	2635.62	1255.39	−1.07	2.06E-207	5.65E-205
**Cactus**						
Unigene66277	654	56.66	126.22	1.16	2.50E-13	1.26E-11
Unigene70354	1227	64.98	188.38	1.54	1.85E-13	9.70E-12
**Pellino**						
Unigene62396	490	50.64	102.46	1.02	3.15E-07	6.14E-06
**Cactin**						
Unigene2785	487	42.80	13.84	−1.63	2.38E-06	3.91E-05
**Caspase**						
Unigene13475	850	17.90	41.23	1.20	1.01E-06	1.78E-05
**Catalase**						
Unigene42771	246	110.28	16.44	−2.75	6.15E-14	3.68E-12

NP, non-parasitization. P, parasitization. RPKM, Reads per kb per million reads. FDR, false discovery rate. FDR ≤0.001 and the absolute value of log2 Ratio ≥1 were used in this study.

In insects, recognition of nonself is the initial process of the innate immune response. Regarding prokaryotic pathogens and fungi, it is notably known that the recognition step is mediated by a group of proteins, known as pattern recognition proteins (PRPs) such as GRP/GNBP, PGRP, and CTL [41]. GRP/GNBP was first discovered in the insect order Lepidoptera (butterflies and moths), which recognizes the β-1,3-glucans of fungal cell walls and contains a C-terminal glycosyl hydrolase 16 (GH16) domain lacking the active site of GH16 [42]. PGRP was first discovered from the hemolymph and cuticle of *Bombyx mori*, which specifically binds to peptidoglycan [43]. CTLs are a group of carbohydrate-binding proteins that share a common structural motif, the carbohydrate recognition domain [44]. These PRPs can specifically recognize sugars on the surface of microorganisms and cause a series of immune responses to effectively resist pathogenic invasions. In invertebrates, they are important components of cellular as well as humoral innate immune responses. They function as pathogen recognition receptors in the innate immune system by activating PPO in hemolymph, promoting the hemocyte phagocytosis, and by participating in hemocyte nodule formation and encapsulation [43], [45]–[48]. In this study, transcriptions of GRP/GNBP, PGRP, and CTL were regulated in parasitized *T. molitor* pupae. Previously studies also support these PRPs as candidates for the recognition function involved in the parasitoid-host interaction. Microarray experiments showed that two PGRPs of *Drosophila* were significantly upregulated after parasitoid attack [49]. *Hyposoter didymator* polydnavirus infection can down-regulate a *Spodoptera frugiperda* gene with high similarity with the *Manduca sexta* immulectin-2 [50]. Especially, it was reported that the gene *lectin*-*24A* was massively up-regulated in parasitized *Drosophila* larvae after parasitization at the time when the capsule was formed [51]. A similar investigation indicated that the inhibitory effect of *P. puparum* venom on host encapsulation is consistent with its effect in suppressing CTL expression [52]. Thus, CLT is an important immune candidate involved in the suppression of host cellular immune defense induced by the parasitoid. The other PRPs altered by parasitization might also be needed for the egg or offspring of parasitoid to avoid or evade the host immune response, which are mediated by the introduction of venomous maternal factor of parasitoid wasps.

For opposing the invasion of foreign agents, a hallmark of the holometabolous insect hosts defense is the challenge-induced synthesis and secretion of potent AMPs that accumulate in the hemolymph [53]. AMP production is an important humoral immune reaction in insects. It plays crucial roles in eliminating infections from various bacteria, fungi, viruses and protozoa by acting as pore-formers or metabolic inhibitors [54]. Eight unigenes encoding putative AMPs were found to be regulated in the parasitized *T. molitor* pupae (Table 4), including attacin (Unigene48479 and Unigene22242), acaloleptin (Unigene8990, Unigene12213, and Unigene15283), lysozyme (Unigene43694 and Unigene46654), and a protein that is similar to antibacterial peptide Cp1 of *T. castaneum* (Unigene61787). Except for lysozyme, the other AMPs were up-regulated. Similarly, few earlier investigations indicated that parasitization induces elevation of AMP transcription in the host. For instance, the parasitoid *Leptopilina boulardi* can trigger the transcription of the genes encoding diptericin and cecropin in *Drosophila* susceptible strain [55]. Nicolas et al. [56] also reported that diptericin and cecropin were either unchanged or minimally induced in *Drosophila* susceptible strain following attack by larval (*L. boulardi* and *L. heterotoma*) and pupal stage (*Trichopria*) parasitoids. In *P. xylostella*, gloverin, moricin, lysozyme II, and cecropin were up-regulated by *D. semiclausum* attack [40]. In contrast, like the lysozyme down-regulated by *S. guani* attack, there were a number of host AMPs that responded similarly when parasitized by parasitoid or injected with parasitoid's maternal factors. For example, transcript levels of cecropin and gloverin in eggs of *M. sexta* were suppressed after parasitization by *Trichogramma evanescens* [57]. After *Pieris rapae* pupae injected with *P. puparum* venom, ten potential antimicrobial molecules including cecropin, lysozyme, attacin, lebocin, proline-rich AMP, cysteine-rich peptide, gallerimycin and immune inducible peptide had lower transcript levels in hemocytes or fat body of the venom injected hosts[52], demonstrating that the transcriptional down regulation of these antimicrobial genes is due to the venom. Nevertheless, parasitization of *Drosophila* resistant strain by the corresponding parasitoids did not induce transcripts of diptericin and cecropin whilst the susceptible strain did [55], [56]. This lack of antibacterial transcript induction in the parasitized resistant strain suggests that the maternal factors injected during oviposition by the parasitoids are not strong elicitors. Also, some AMPs of lepidopteran larvae and pupae such as moricin and gloverin were found to be not inducible by bacterial challenge [58], [59], while they were appeared to be in response to parasitoid attack [40], [57]. Accordingly, we would speculate that the induction of AMP following parasitization might be different from that case of bacterial. A specific genetic factor (or highly linked factors), designated humoral response to parasitoid (hrtp) in *D. melanogaster*, has been found to be essential for the activation of diptericin by the parasitoid wasp [60], indicating that the expression of antibacterial gene after parasitoid infection is linked to a genetic regulation. However, it remains confusing that why the AMPs were differentially regulated in the same or different parasitoid-host systems, e.g., lysozyme in the hosts up-regulated by *D. semiclausum* whilst down-regulated by *S. guani* in this study. Furthermore, since parasitoid attack involves wounding and penetration, parasitized insects are subsequently more susceptible to opportunistic infections. It is possible that the regulation of antimicrobial peptides is associated with damage to the exoskeleton and low-level exposure to microbial factors on the surface of the host or ovipositor of the wasp [51].

In response to invaders, an essential component of the cell-mediated immediate immune response in insects is the melanization reaction observed at the site of cuticular injury or on the surface of parasites invading the hemocoel [61]. Melanization is the result of proteolytic cascade triggered by minute amounts of elicitors, which is associated with a number of vital proteins such as PPO, SP, SPH, and serpin [62]. It is well documented that parasitoid can survive despite hosts melanotic encapsulation, or destroy with no evidence of this host response [63]. SP, SPH and serpin that retain the ability to inhibit melanization have been indentified from the venom of parasitoids [15]. The inhibition is most likely due to interference with the proteolytic cascade that leads to the activation of PPO by these venom proteins. In the current study, we found that the transcripts of PPO, SP or SPH and serpin were changed after parasitism in *T. molitor*. In addition to transcription, these genes can be regulated at the translational level. In *P. xylostella* larvae parasitized by *Cotesia plutellae*, the expression of pxSerpin 2 gene increased as parasitism progressed, while its protein profile was reduced compared to that of the control, and continued to decrease with the progress of parasitism [64]. Genes with altered expression that are associated with the PPO system are often observed following parasitization [65]–[68]. This should result in a general decrease in PO activation and inhibition of melanization observed in parasitized hosts. Thus, the regulation of these genes upon immune challenge in parasitized host could be part of a parasitoid immune suppressive strategy. It has been reported that the transcripts of PPO in *Spodoptera frugiperda* were differentially affected by *Hyposoter didymator* Ichnovirus and *Microplitis demolitor* Bracovirus [69].

Reactive oxygen species (ROS) production is an immediate acute-phase oxidative defense in response to pathogen assault or cellular stress such as phagocytosis and melanotic encapsulation [70]. Due to the cytotoxicity of ROS, its production is tightly regulated by immune responsive antioxidant enzymes such as SOD, catalase, glutathione oxidase, thioredoxin reductase, and peroxidase [71]. Our analysis shows that the transcript levels of SOD, catalase, and peroxiredoxin (belonging to peroxidase) genes were altered after parasitization by *S. guani*. Moreover, two peroxidases, thiol peroxiredoxin and peroxiredoxin were strongly induced in the plasma of *Papilio xuthus* after parasitization [72]. However, the expression change may cause perturbation in normal SOD and peroxiredoxin function, which would lead to the production of parasitoid egg or offspring killing by cytotoxic ROS. Supposedly, the excessive produced ROS might be sequestered and localized to the surface of the parasitoid, thereby preventing adverse systemic reactions from occurring in the open circulatory system [48]. Given the diverse roles of ROS in immune defense, altered transcription of these genes following parasitization could be an epithelial host response to the physiological injury brought about during the process of oviposition or an alteration that adversely affects metabolism.

Hexamerins consist of six identical or similar subunits with a molecular mass in the range of 80 kDa each [73]. They belong to a protein superfamily that also comprises arthropod PPOs and hemocyanins, crustacean pseudohemocyanins, and the hexamerin receptors discovered in the diptera [74]. In insects, as hexamerins have been found at very high concentrations in the hemolymph of many insect species, they are thought to act mainly as storage proteins in non-feeding periods [75]. Among the identified immune related DGEs, six unigenes encoding hexamerin (one up-regulate and five down-regulated) were found. Similarly, the transcriptional responses of hexamerins to parasitization have been documented in several host-parasitoid systems with different regulation strategies [76]–[79]. Although distinct regulatory mechanisms at transcriptional level may exist in different host–parasitoid systems, studies so far indicate that the titer of hexamerins in the hemolymph increase in the hosts following parasitism [80]–[83]. Regardless of the critical role of hexamerins acting as storage proteins in growth and development, it has been evidenced that some hexamerins are part of the innate immune system in various arthropods, acting as pro-coagulants [84]. They also fulfill immune functions in insects. For example, it was demonstrated that the expression of genes and proteins in the honey bee was significantly changed after activation of the immune system by bacterial challenge or even after injury caused by injection of water [85], [86]. It has been speculated that arylphorin suppresses hemocyte degranulation and subsequent immune reactions that lead to the encapsulation of the parasitoid's egg [87]. The high content of aromatic amino acids in the arylphorins may enhance the cross-linking capabilities of this protein to form ideal clotting, thereby isolating parasitoid egg from host immune response or reducing host humoral immune reaction cascade [73]. Based on these hypotheses, hexamerins are probably related to some function in host protection, while much work still needs to be done to explore the mechanisms.

The innate immune system of insects relies on both humoral and cellular immune responses that are triggered by the immune challenge and mediated via activation of signaling pathways [88]. Four signal transduction pathways, Toll, Imd, JNK and JAK/STAT, are known to be present in insect immunity [89]. On the other hand, the immune repertoire genes participating in the signal transduction pathways have remained highly conserved throughout the different insect orders [36]. The expression of most of these genes is switched on following invasion of microbes, but the signaling pathways involved in anti-parasite responses are not well- understood [90]. Our results indicated that several transcripts associated with Toll and Imd pathway including cactus, pellino, cactin, and caspase were affected after *S. guani* attack. Similarly, even though the transcripts of most components of the different signaling pathways were found in the transcriptome database of *P. xylostella*, only transcription levels of proteins that showed similarity to the Toll receptor were up-regulated after parasitoid attack [40]. In addition to the components of Toll and Imd pathways, microarray-based genome-wide analyses indicated that parasitism by *Asobara tabida* or *L. boulardi*, but not *L. heterotoma* can induce multiple genes encoding signaling components of the JAK/STAT pathway in *Drosophila* [49], [51]. The data confirmed that the signaling pathways play a role in the anti-parasitoid immune response. It is likely that a deactivation of the signaling cascade might be directly linked to a suppression of host immune defense by the parasitoid.

### Conclusion

In summary, the transcriptome of *T. molitor* pupae was sequenced with massive parallel pyrosequencing using Illumina sequencing technology, permitting the discovery of a great number of genes involved in immunity. The transcriptome profiling data sets obtained in this study make a significant contribution to existing sequence resources for the beetle. The explored immune genes provide valuable resource for future research in understanding immune system and defense mechanisms of *T. molitor* as well as many other Coleopteran species. Additionally, we investigated the global gene transcription profiles of *T. molitor* in response to parasitization by *S. guani*, especially with focus on the immune aspect. A large number of *T. molitor* genes, including immune-related gene, were differentially expressed after parasitization by *S. guani*. It is noteworthy that parasitization induces sets of genes that had not previously been implicated in immune function, or are not activated upon bacterial infection. This provides new insight into the host immune defense mechanisms against parasitoid, and the ways in which parasitoids overcome these mechanisms from a molecular prospective.

## Supporting Information

Figure S1 **Length distribution of** ***Tenebrio molitor*** **contigs.** Horizontal axis represents the length of contigs and vertical axis represents number of contigs.
(TIF)

Figure S2 **Length distribution of** ***Tenebrio molitor*** **scaffolds.** Horizontal axis represents the length of scaffolds and vertical axis represents number of scaffolds.
(TIF)

Figure S3 **Classification of raw reads in non-parasitized (NP) and parasitized (P)** ***Tenebrio molitor*** **pupae.** Numbers in parentheses show the percentage of each type of read among the total raw reads.
(TIF)

Figure S4 **Distribution of distinct clean reads in non-parasitized (NP) and parasitized (P)** ***Tenebrio molitor*** **pupae.** Numbers in the square brackets indicate the range of copy numbers for a specific category of reads. The data in parentheses indicate the percentage of corresponding reads among the total distinct reads.
(TIF)

Figure S5 **Distribution of gene coverage in non-parasitized (NP) and parasitized (P)** ***Tenebrio molitor*** **pupae.**
(TIF)

Table S1 **Primers used in qRT-PCR for validating differentially expressed genes.**
(XLS)

Table S2 **Top Blast hits from NCBI nr database. BLASTX against the nr protein database was used with a cutoff E-value of 10^−5^.**
(XLS)

Table S3 **Gene ontology (GO) annotation of unigenes.**
(XLS)

Table S4 **Kyoto Encyclopedia of Genes and Genome (KEGG) annotation of unigenes.**
(XLS)

Table S5 **Immunity-related genes identified in** ***Tenebrio molitor*** **transcriptome.**
(XLS)

Table S6 **Differentially expressed genes between non-parasitized (NP) and parasitized (P)** ***Tenebrio molitor*** **pupae.** Raw intensity, the total number of reads sequenced for each gene. RPKM, Reads per kb per million reads. FDR, false discovery rate. FDR ≤0.001 and the absolute value of log2Ratio ≥1 were used in this study.
(XLS)

Table S7 **Description of the top 20 most up- and down-regulated genes.**
(XLS)

Table S8 **Gene Ontology (GO) enrichment analysis of differentially expressed genes.**
(XLS)

Table S9 **Kyoto Encyclopedia of Genes and Genome (KEGG) enrichment analysis of differentially expressed genes.**
(XLS)
