# Supplementary figures and images for: Transcriptomic Immune Response of Tenebrio molitor Pupae to Parasitization by Scleroderma guani
Source: PLoS One. 2013 Jan 14;8(1):e54411. doi: 10.1371/journal.pone.0054411 (PMC3544796; doi:10.1371/journal.pone.0054411)

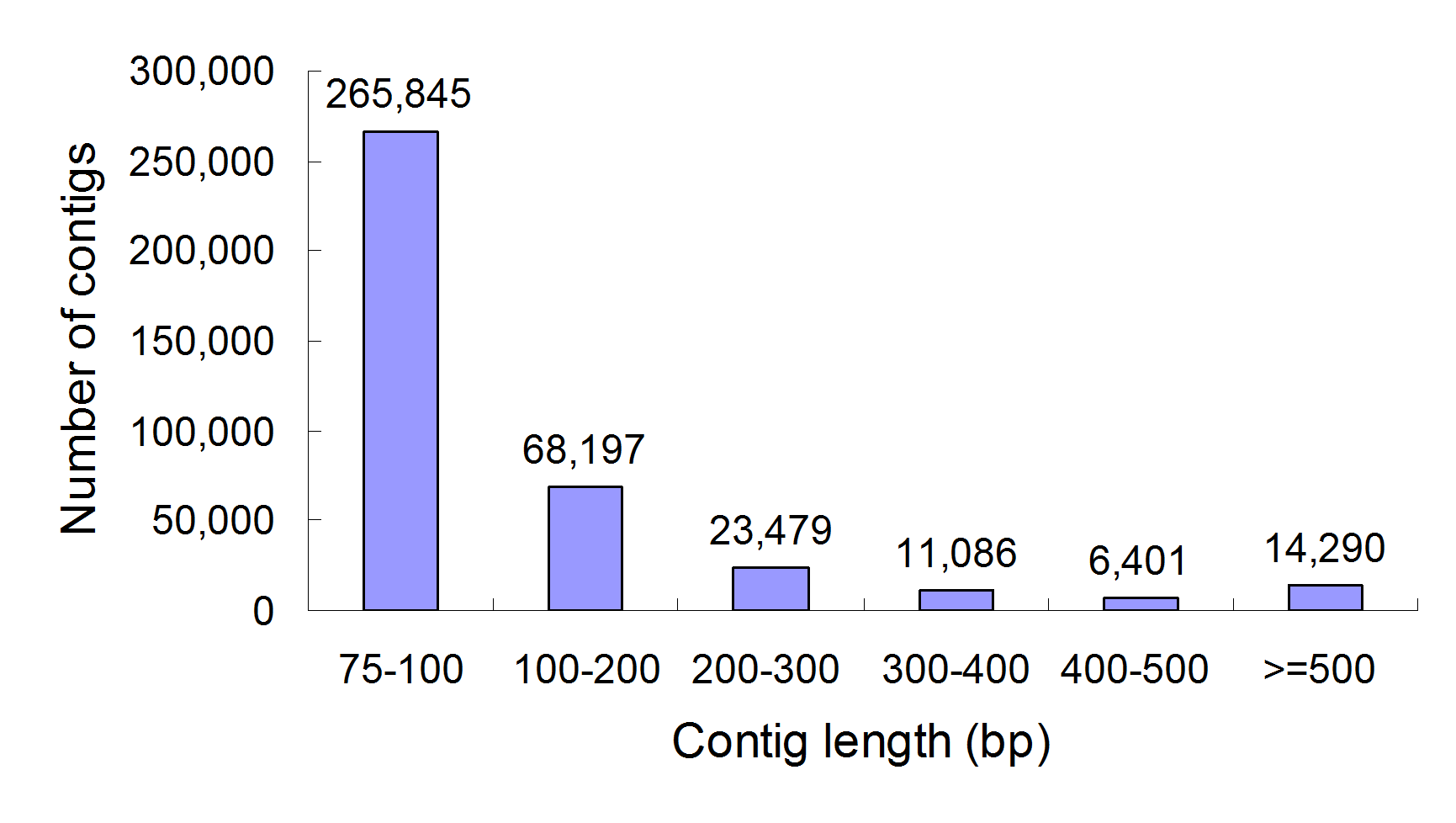

Supplement: Figure S1 — Length distribution of Tenebrio molitor contigs. Horizontal axis represents the length of contigs and vertical axis represents number of contigs. (TIF) [file pone.0054411.s001.tif]

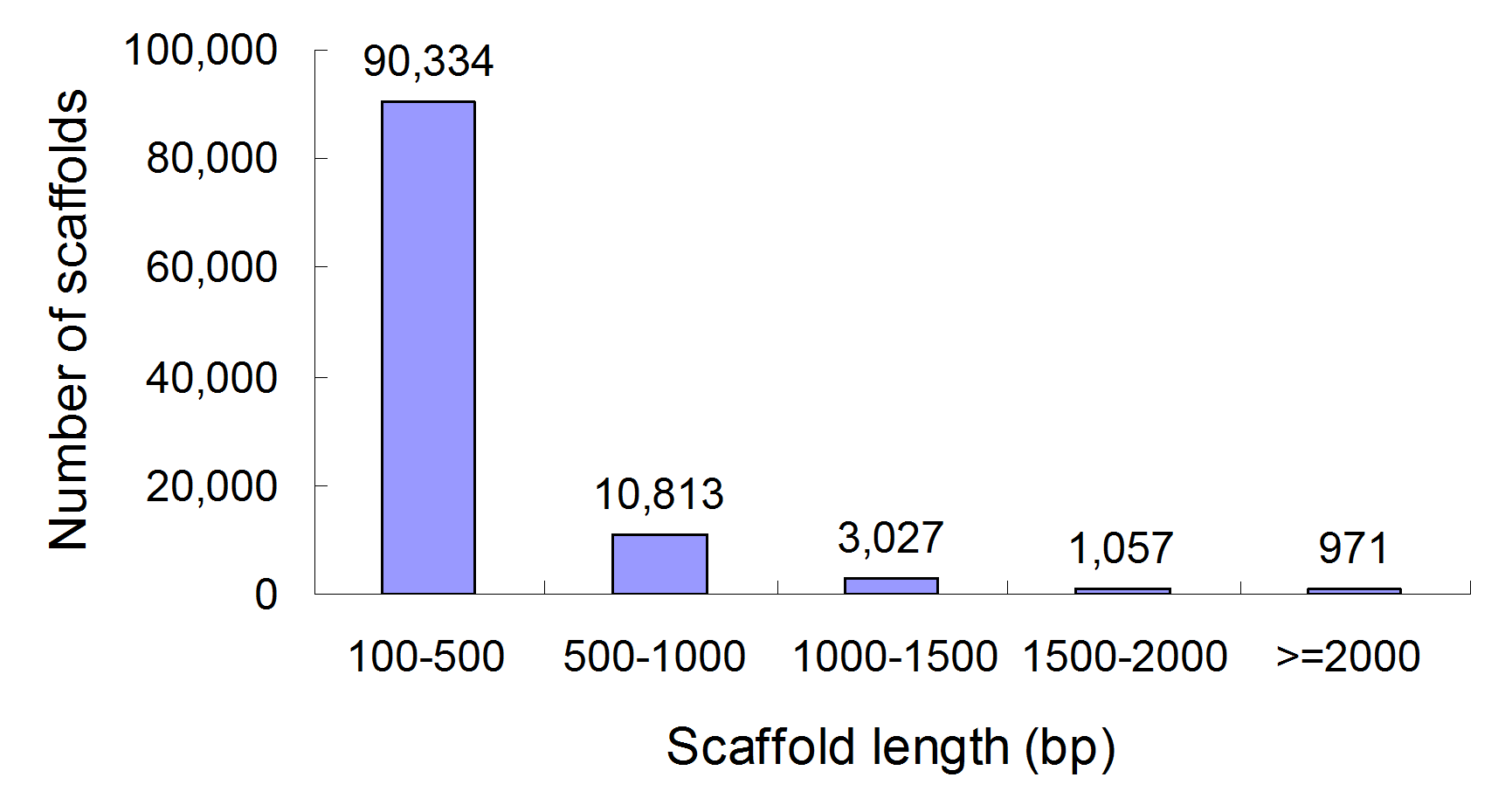

Supplement: Figure S2 — Length distribution of Tenebrio molitor scaffolds. Horizontal axis represents the length of scaffolds and vertical axis represents number of scaffolds. (TIF) [file pone.0054411.s002.tif]

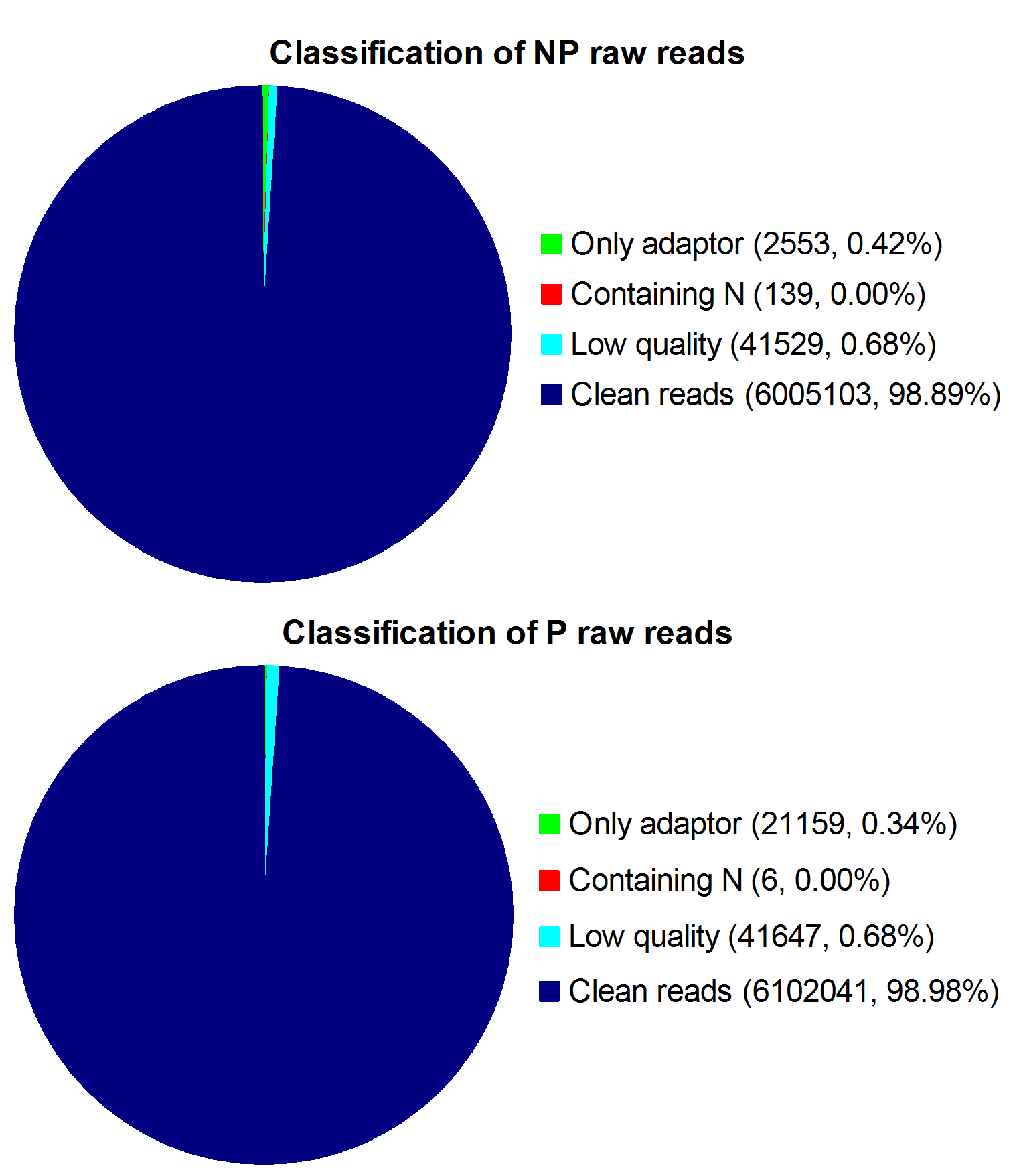

Supplement: Figure S3 — Classification of raw reads in non-parasitized (NP) and parasitized (P) Tenebrio molitor pupae. Numbers in parentheses show the percentage of each type of read among the total raw reads. (TIF) [file pone.0054411.s003.tif]

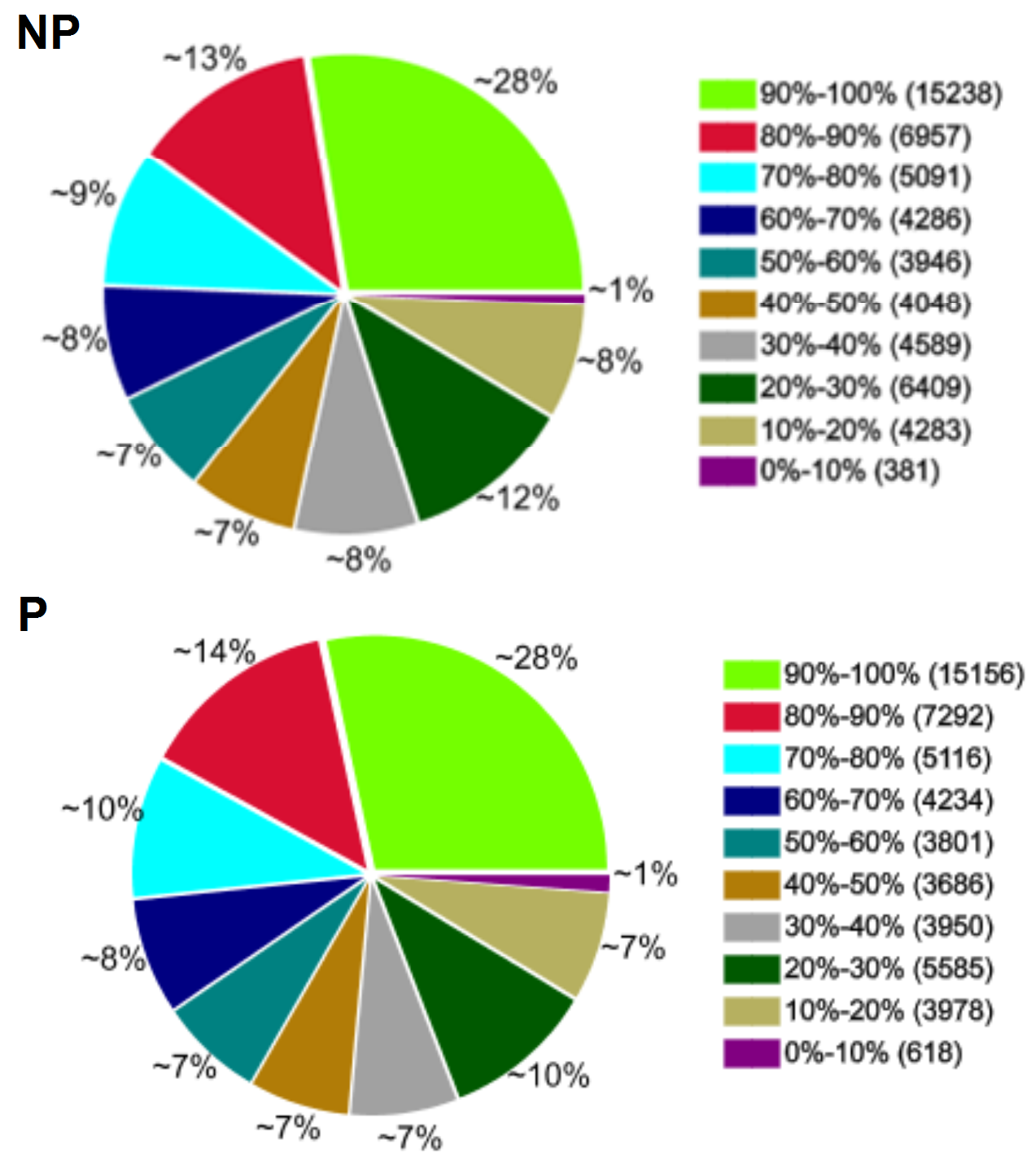

Supplement: Figure S4 — Distribution of distinct clean reads in non-parasitized (NP) and parasitized (P) Tenebrio molitor pupae. Numbers in the square brackets indicate the range of copy numbers for a specific category of reads. The data in parentheses indicate the percentage of corresponding reads among the total distinct reads. (TIF) [file pone.0054411.s004.tif]

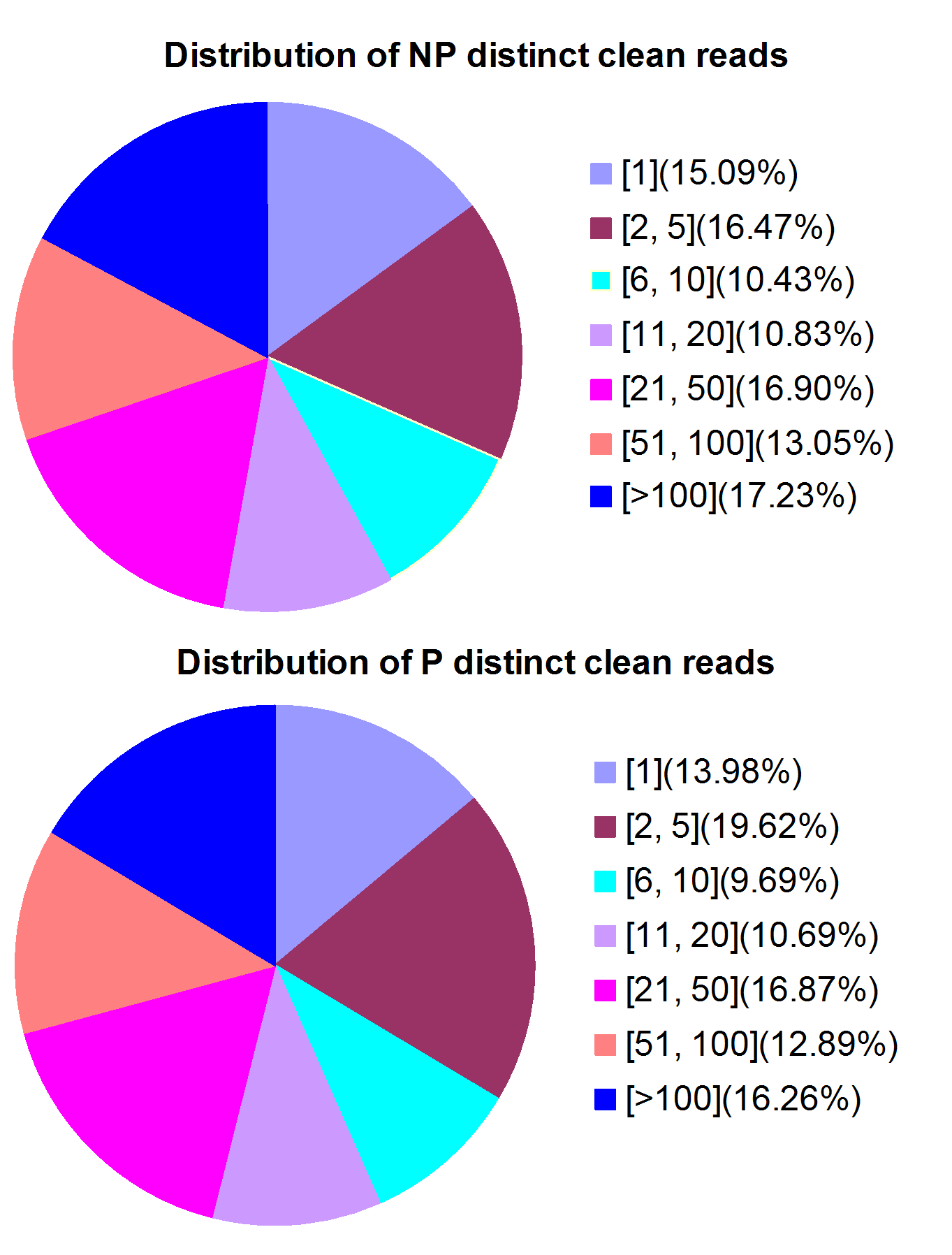

Supplement: Figure S5 — Distribution of gene coverage in non-parasitized (NP) and parasitized (P) Tenebrio molitor pupae. (TIF) [file pone.0054411.s005.tif]
